# Utility of patient decision aids (PDA) in stress urinary incontinence surgery

**DOI:** 10.1007/s00192-019-03982-1

**Published:** 2019-06-01

**Authors:** Swati Jha, Jonathan Duckett

**Affiliations:** 1 Department of Urogynaecology, Sheffield Teaching Hospitals NHS Foundation Trust, Jessop Wing, Tree Root Walk, Sheffield, S10 2SF UK; 2 Department of Obstetrics and Gynaecology, Medway Hospital, Windmill Rd, Gillingham, Kent ME7 5NY UK

**Keywords:** Patient decision aid, PDA, Stress incontinence surgery, Shared decision making, Decision conflict

## Abstract

**Introduction and hypothesis:**

Patient decision aids (PDAs) facilitate shared decision making allowing patients to make decisions about their healthcare that take into account their personal values and preferences. The aim of this study was to establish whether a PDA used in women requiring stress incontinence surgery is helpful to women when making choices about the treatments they choose by using a Decision Conflict Scale (DCS).

**Methods:**

Forty-five consecutive women were identified as having stress urinary incontinence and had completed all conservative treatments. All patients included in the study had stress urinary incontinence confirmed on urodynamic testing and were given the PDA at the point where they needed to make a decision about surgery. Following completion of the PDA, patients were given a DCS to complete which measures personal perceptions of uncertainty when making a decision about treatment.

**Results:**

Forty-three out of 45 (95.5%) patients scored 4/4 for the DCS indicating they were sure of their decision. Two patients (4.5%) scored 3/4 and were therefore unsure of their choice. No patient scored < 3 on the DCS. The choice of procedures varied in all the ages and two women opted to have no treatment.

**Conclusions:**

The use of a PDA in the surgical treatment of stress urinary incontinence reduces decision conflict and ensures patients are sure of their decision, understand the information provided as well as the risk benefit ratio of the various options and feel they have adequate support and advice to make a choice.

**Electronic supplementary material:**

The online version of this article (10.1007/s00192-019-03982-1) contains supplementary material, which is available to authorized users.

## Introduction

The consent process is an exchange of information between the doctor and patient which needs to be tailored to the individual circumstances. However, it is important not to make assumptions about the amount of information a patient may want, the factors they may consider significant and the level of knowledge of the proposed treatment.

For consent to be fully informed it is advised that wherever possible patients should be involved in the decision-making process. In accordance with General Medical Council guidance on Consent [1], doctors use their specialist knowledge and expertise to identify which investigations and treatments are likely to be of overall benefit. These options are then explained to the patient whilst highlighting the potential benefits, risks, burdens and side effects of each option. When informing patients about the various treatments it is important to inform them of any uncertainties including the likely success and failure. It is also important to highlight the option of no treatment. It is then the patient’s prerogative to weigh up the different options and decide whether they wish to accept any of the options or opt for no active treatment.

When sharing information with patients this should be done in a manner that patients can understand. Patients should be allowed an opportunity to reflect on the options before they make a decision, particularly where the information is complex and the treatment has considerable risks. This process of shared decision making encourages active participation by patients in their healthcare decisions. This can be further facilitated by the use of patient decision aids (PDAs).

PDAs allow patients to make informed decisions about healthcare choices taking into account their personal values and preferences [2]. This can be for both diagnostic and therapeutic procedures. They make it easier for patients and clinicians to discuss treatment options [3–5]. A PDA has the following objectives:

- Inform patients of the evidence base to the available options;
- Enable patients to identify what is important to them so that their choices reflect their preferences and values;
- Encourage active participation by the patient in the decision-making process.

The aim of this study was to establish whether a PDA used in women requiring stress incontinence surgery is helpful to women when making choices about the treatments they choose by using a Decision Conflict Scale.

## Methods

This study was conducted in two units using a PDA developed for women scheduled to undergo stress incontinence surgery. Data were collected over a 12-month period (March 2018–February 2019). At both units where the authors work, all patients were offered all four surgical choices for stress urinary incontinence including bulking agents, colposuspension, fascial slings and synthetic midurethral tapes. Patient information leaflets developed by IUGA (International Urogynaecology Association) and BSUG (British Society of Urogynaecology) were used and leaflets on all the procedures were provided to all the patients when providing them with the PDA. The counselling in all cases was by the two authors. All patients agreed to work through the PDA.

The development of the PDA went through the usual processes described for the development of these tools and both patients and clinicians had input into this process. This included the following steps:

1. Scoping and design: The content was guided by a needs analysis, scientific evidence and guidelines for the management of stress urinary incontinence.
2. Alpha testing with patients and clinicians in an iterative process: this involved testing the comprehensibility and usability of the PDA by volunteers willing to participate.
3. Beta testing in real-life conditions: the feasibility was testing including by individuals who were not involved in the design of the PDA.

The PDA was developed in accordance with the Ottawa Decision Support Framework [6]. This is based on expectancy value, decisional conflict and social support theories. This framework includes three key components:

1. assessment of determinants of decisions for both patients and clinicians;
2. provision of decision support interventions to prepare the patient and clinician to make and implement a decision;
3. evaluation of the success of the interventions at improving the quality and outcomes of the decision process.

Further details of the development of the PDA will not be discussed in this article. The PDA used for this study is available as Appendix 1.

For the purposes of this study, the utility of the PDA in clinical practice was tested in the beta testing phase.

The tool used to assess the utility of the PDA in clinical practice was the Decision Conflict Scale (DCS). DCS measures personal perceptions of uncertainty when making a decision about treatment. It takes into account the modifiable factors contributing to uncertainty such as feeling uninformed or being unclear about personal values or even feeling unsupported in decision making. It also assesses feelings of whether the choice is informed, values based and likely to be implemented to result in satisfaction. The degree of decisional conflict can be reduced with the use of decision support interventions such as the PDA. There are significant data to support the effects of using a PDA on decisional conflict [7].

There are four versions of the DCS [8]. One is recommended for use in clinical practice and the remaining three are research tools. The version used for the purposes of this study was the clinical version, also referred to as the “SURE” test version [9]. This has been validated for use in clinical practice. This has four items and two response categories and is shown in Table 1 with the responses obtained.

**Table 1:** Decision conflict scale

		Response (yes/no)
Sure of myself	Do you feel SURE about the best choice for you?	43/2
Understanding information	Do you know the benefits and risks of each option?	45/0
Risk benefit ratio	Are you clear about which benefits and risks matter to you most?	45/0
Encouragement	Do you have enough support and advice to make a choice?	45/0

Items are scored as “1” for “yes” and “0” for “no”. Score ranges can be from 0 indicating extremely high decisional conflict to 4 indicating no decisional conflict. A score ≤ 3 indicates decisional conflict whereas a score of 4 indicates no decisional conflict.

On this basis, assuming a one-point difference is of clinical and practical importance, then to have an 80% power of detecting a 1-point mean difference in the DCS at the 5% (two-sided) level would require 35 patients.

The age and demographic data of the women completing the study were analysed. All women included in this study were able to speak and understand English as their first language and had completed conservative treatments for their stress urinary incontinence. All women included in the study had urodynamic proven stress urinary incontinence and were given the PDA to enable them to make an informed decision about their choice of surgery. They were then invited back to the outpatient department to inform the clinician of their decision. At this point they were given the DCS to complete to see how beneficial the PDA had been to their decision-making process.

A few months after the introduction of the PDA the IMMDR (Independent Medicines and Medical Devices Review), referred to as the Cumberlege Review, implemented a pause on the use of vaginal mesh for incontinence in the UK [10]. Patients and clinicians were not made aware of this pause beforehand. Following the pause, though the synthetic tape option was no longer available, it was still on the PDA. As part of this survey we assessed whether women’s preferred choice of surgical procedure changed after the pause was implemented. All patients were discussed at the local Urogynaecology MDT. For the cases reported here, there was no conflict between the patient’s choice of surgery and the MDT consensus to their choice, but these were all cases of primary incontinence surgery.

This was conducted as a service evaluation so formal ethical approval was not required.

## Results

Forty-five consecutive women were given the PDA prior to stress urinary incontinence surgery. The mean age of the women was 48.27 (range 29–72 years) and all women were white Caucasians who spoke English as their first language.

Forty-three out of 45 (95.5%) patients scored 4/4 for the DCS and responded with a “yes” to all four items. Two patients (4.5%) scored 3/4 and were unsure of their choice even though they responded in the affirmative to the other items. No patient scored < 3 on the DCS.

The choice of procedure made by patients is shown in Table 2. Two patients opted for no surgical treatment after receiving the PDA. Both these patients scored 4/4 on the DCS indicating no decisional conflict, i.e., they were sure of their decision not to have treatment.

**Table 2:** Choice of procedure

Procedure	*N* (%)	*N* before pause	*N* after pause
Bulking agent	13 (29)	7	6
Colposuspension	10 (22)	2	8
Fascial sling	6 (13)	4	2
Synthetic tape	14 (31)	13	1
None	2 (4)	NA	NA

Age seemed to have no bearing on the surgical procedure women chose.

Before the implementation of the vaginal mesh pause 50% (13/26) women opted for synthetic tapes, but after the pause this was only 5%(1/19). This patient has opted to wait for pause reversal rather than proceed with any of the other options. The rates of the different procedures before and after the UK vaginal mesh pause are shown in Table 2. Both women who opted for no treatment did so after the implementation of the vaginal mesh pause.

## Discussion

The use of a PDA in the surgical treatment of stress urinary incontinence reduces decision conflict to a minimum by ensuring patients are sure of their decision, understand the information provided and the risk-benefit ratio of the various options as well as feeling they have adequate support and advice to make a choice. Age does not impact on the surgical choice women make. The implementation of a pause on the use of synthetic mid-urethral tapes appears to have altered the preferred choice of surgical procedure for stress urinary incontinence. However, as the pause coincides with recent adverse publicity for the use of mesh, it is difficult to know whether this reduction was partly attributable to this adverse media attention. It is also possible that the counselling offered had an impact on patient choice as clinicians were unable to perform the synthetic tape even if patients chose it.

This is the first study analysing the utility of a PDA in clinical practice for stress urinary incontinence surgery using a formal tool to assess conflict score. The DCS has shown promise for screening for decisional conflict in both French- and English-speaking patients. It has been validated for use and shown to have both reliability and validity when used in practice [9] and has been used in other studies to assess utility of a PDA in practice.

This survey suggests that using a PDA is useful in ensuring patients are certain about their choices when choosing SUI surgery. This is encouraging for clinicians and has the potential to affect future practice in this area. As this survey only reports on data after the implementation of the PDA, it is difficult to be certain that there has been a positive impact on decision making as there is no comparator either before the introduction of the PDA or from analysis of a cohort of patients who did not receive the PDA. The results can only be extrapolated to white Caucasian English-speaking women as the study cohort included this group.

In a study by Spyroulis et al. [11] using a PDA designed for stress urinary incontinence surgery, it was proposed that there was better patient understanding of their values and choices, and patients felt better informed and better valued. Their study cohort included 30 women and they did not test the utility of the PDA using a formal tool. Their study analysed patient values as derived from the PDA instead.

PDAs have been used across a wide range of conditions for screening, diagnostics and therapy. In a Cochrane systematic review [4] analysing the impact of PDAs on a wide range of conditions, it was shown that they decreased decisional conflict related to feeling uninformed (MD -9.28/100; 95% CI -12.20 to −6.36; 27 studies; *N* = 5707; high-quality studies; *N* = 5068; high-quality evidence), and the proportion of people who were passive in decision making (RR 0.68; 95% CI 0.55 to 0.83; 16 studies; *N* = 3180; moderate-quality evidence). These were used in a range of health treatment and screening decisions and were found to reduce the proportion of undecided participants. PDAs also appeared to have a positive effect on patient-clinician communication. In addition exposure to a decision aid improved satisfaction with their decision, the decision-making process and/or the preparation for decision making compared with usual care. This survey shows that in the context of SUI surgery, a PDA has the same benefits as its use in other clinical scenarios.

Introducing PDA in practice has significant implications for the clinical consultation, especially the duration [12]. In a Cochrane systematic review [4] of PDA, a variable impact on the length of the clinical consultation was shown, ranging from reducing the consultation by 8 min to increasing it by 23 min compared with a standard consultation (median + 2.5 min).

Over the past few years there have been significant concerns about the safety of continence surgery. There have also been many court actions and increasingly clinicians are being successfully sued for failing to inform patients adequately of all options prior to surgery. The Montgomery [13] ruling of 2015 drew fresh attention to informed consent. The use of a PDA ensures that the consent process is Montgomery compliant and that all material risks to which a reasonable person would attach significance have been discussed. This survey also lends credence to the hypothesis that the use of a PDA allows a patient to be sure of their decision and therefore take responsibility for the choices.

It is unclear whether clinicians’ personal preferences impact the choices patients make following use of the PDA. In addition, if a clinician is unable to offer all the choices for surgery, it is unclear whether a patient’s decision making would be influenced by this. The impact of the pause has been a proxy for this situation and indicates that where all options are not available, patients choose alternatives. Further research is needed to investigate these areas of uncertainty.

## Electronic supplementary material

ESM 1 (DOC 208 kb)
