# Supplementary material for: Utility of patient decision aids (PDA) in stress urinary incontinence surgery
Source: Int Urogynecol J. 2019 Jun 1;30(9):1483–6. doi: 10.1007/s00192-019-03982-1 (PMC6706361; doi:10.1007/s00192-019-03982-1)
Supplement: Supplementary file 1 — (DOC 208 kb) [file 192_2019_3982_MOESM1_ESM.doc]

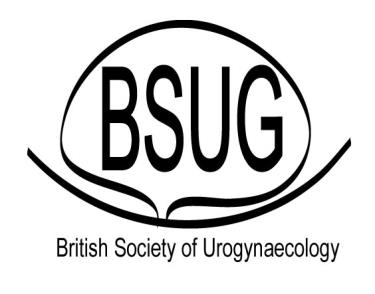


Patient

Label

**Patient Decision Aid (PDA): Choosing Surgery for Stress Urinary Incontinence**

Shared-Decision Making Tool

**PLEASE COMPLETE SECTIONS C and E DURING OR AFTER THOROUGH DISCUSSION WITH YOUR HEALTHCARE PROFESSIONAL AND RETURN THIS FORM TO A MEMBER OF STAFF OR BY POST TO ……………………………………………**


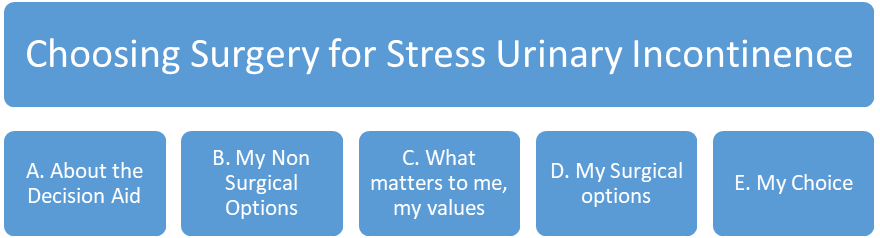


1. About this decision aid

This decision aid aims to help you make a choice when you are considering surgical options for stress urinary incontinence after non-surgical options have been tried for a reasonable length of time without success. It is important that the surgical option chosen is personalised for you. As well as being as safe and effective as possible, the type of surgery chosen will focus on your individual needs and preferences as much as possible.

By finding out more about you we can improve shared-decision making and subsequently the overall outcome of treatment. One way to make this better is for your healthcare professional to find out what is important to you. During decision-making, it is important to establish *‘what matters to you’*.

**Once you have read this document and discussed your options with your healthcare professional, you will be able to complete sections C and E with their support.**

**Completed forms should be handed back to the team looking after you or returned by post.**

**What happens after I complete this form?**

Your choices will be discussed at our dedicated meeting (multidisciplinary team or MDT meeting) to help plan your care. You will be informed of the outcome of the discussions, including any recommendations for you to consider.

**What if I do not want to give the information?**

You do not need to share any information that you do not want to.

We will be happy to help you with any concerns you may have at any stage.

**Can I change the information once I have completed it?**

Yes, we recognise that *what matters to you* may change during the decision-making process. You can change your information and decision at any time.

B) What are the non-surgical options for treatment of my Stress Urinary Incontinence (SUI)?

- If you have **urinary** leakage on coughing, sneezing or physical activities various treatment options are available however you may choose to have no treatment.

**Lifestyle Changes**

- Review of your diet and fluid intake
- Weight loss programme if you are overweight
- Referral to continence nurse for bladder training and further advice

**Pelvic Floor Muscle Training (PFMT)**

- You should be offered a PFMT programme by a specialist, usually a physiotherapist before considering surgery.
- PFMT can also be useful if you have other urinary symptoms e.g. urgency.

**Medical Treatment**

- You may consider a trial of Duloxetine therapy (tablets that help SUI). These only work whilst they are being taken.
- Your health care professional should discuss the potential side effects and duration of this treatment.

**Vaginal Devices**

- You may consider a continence pessary or device if suitable.

YOU ARE HERE

- Surgery should only be considered if the above treatment options have not improved your symptoms enough or you have decided that you do not wish to try them.
- Your individual case will be discussed at the multidisciplinary team (MDT) meeting and

your healthcare professional will explain this to you. At the MDT your medical notes and the results of any tests are reviewed. This meeting is attended by urogynaecologists, specialist nurses, physiotherapists and urologists

***Please ask your health care professional for the leaflet about the specific surgical procedure(s) you are considering.***

***Referral to a different clinician (or a different hospital) may be required, depending on availability of expertise at your local hospital.***

1. What matters to me? (PLEASE COMPLETE)

- Please let us know what is important to you from the list below.
- Some things that matter to you may be physical, psychological, emotional or social.
- There are no “right or wrong” answers as it is about you.
- A member of staff can help you to complete it and provide additional information.

***Please rate from 0 to 10 (0 low priority, 10 high priority) next to each of the following items:***

| **What matters to you** | **Importance out of 10** | **Top 3**  **(Please tick)** |
| --- | --- | --- |
| - Cure of leakage | **0** 1 2 3 4 5 6 7 8 9 **10** |  |
| - Using fewer continence pads | **0** 1 2 3 4 5 6 7 8 9 **10** |  |
| - Avoiding a hospital stay | **0** 1 2 3 4 5 6 7 8 9 **10** |  |
| - Shorter hospital stay | **0** 1 2 3 4 5 6 7 8 9 **10** |  |
| - Quick recovery and return to normal activities | **0** 1 2 3 4 5 6 7 8 9 **10** |  |
| - Avoiding major abdominal surgery | **0** 1 2 3 4 5 6 7 8 9 **10** |  |
| - Avoiding repeat surgery in the future | **0** 1 2 3 4 5 6 7 8 9 **10** |  |
| - Whether you have plans for a pregnancy in the future | **0** 1 2 3 4 5 6 7 8 9 **10** |  |
| - Less pain after surgery | **0** 1 2 3 4 5 6 7 8 9 **10** |  |
| - Avoiding a synthetic mesh tape and its complications | **0** 1 2 3 4 5 6 7 8 9 **10** |  |
| - Avoiding self-catheterisation | **0** 1 2 3 4 5 6 7 8 9 **10** |  |
| - Avoiding general anaesthesia | **0** 1 2 3 4 5 6 7 8 9 **10** |  |
| - Avoiding local anaesthesia | **0** 1 2 3 4 5 6 7 8 9 **10** |  |
| - Others (what are they) | **0** 1 2 3 4 5 6 7 8 9 **10** |  |

1. My surgical options

Table below compares the main advantages and disadvantages of the four surgical procedures for treatment of stress urinary incontinence:

|  | **Colposuspension** | **Synthetic Vaginal Mesh**  **Tape** | **Fascial Sling (Natural Tissue)** | **Urethral Bulking agents** |
| --- | --- | --- | --- | --- |
| What the procedure involves | Surgery where the neck of the bladder is lifted upwards and stitched in place.  This is done through an open tummy cut or by a (laparoscopic) keyhole procedure. | A synthetic mesh tape is inserted to support the urethra (tube carrying urine from the bladder to the outside).This is permanent and not intended for removal.  This is done through a small cut in the vagina. | Surgery where a sling of your own tissues is inserted around the neck of the bladder to support it.  This is done through an open tummy cut. | Surgery where a substance is injected into the walls of the urethra to increase its size (bulk it out) and allow it to remain closed with more force.  No cuts, but done using a syringe. |
| Success in curing or improving incontinence | 80-90% | 80-90% | 80-90% | 50-70% |
| Hospital Stay | 1-4 days in hospital | Usually day case | 2-5 days in hospital | Day case procedure/ outpatient procedure |
| Recovery | 6 weeks | 2-4 weeks | 6 weeks | 2-5 days |
| Anaesthetic | Usually General | General/Spinal/Local with sedation | Usually General | Usually local, but can be general or spinal |
| Immediate complications   - bleeding, blood transfusion, infection, embolism - bladder injury | 5% | 5% | 5% | <1% |
| Mesh complications (removal required) | Not applicable | 3.3% | Not applicable | Not applicable |
| New Prolapse | Up to 10% | Not applicable | Not applicable | Not applicable |
| Stitch Problems | <1% | Not applicable | Not applicable | Not applicable |
| Hernia from tummy cut | <1% | Not applicable | Up to 10% | Not applicable |
| Problems emptying the bladder and need for self-catheterization long term | Up to 5% | Up to 5% | Up to 10% | <1% |
| Urinary Urgency problems | Up to 7% | Up to 7% | Up to 10% | Not applicable |
| Sexual Function deterioration | Up to 10% | Up to 10% | Up to10% | Not applicable |
| What if it doesn’t work? | Repeat surgery carries increased risks, technical difficulties and is less successful. | Repeat surgery carries increased risks, technical difficulties and is less successful. | Repeat surgery carries increased risks, technical difficulties and is less successful. | Repeat surgery is usually safe and this is unlikely to impact on success of other future surgery. |

1. My Choice (PLEASE COMPLETE THIS TABLE)

| **Procedure** | **I will choose this option because…** | **I will NOT choose this option because…** |
| --- | --- | --- |
| **Colposuspension** |  |  |
| **Synthetic Vaginal Mesh Tape** |  |  |
| **Natural Tissue Sling** |  |  |
| **Urethral bulking agent injection** |  |  |

Your signature: ………………………………………………… Your name: …………………….………………………… Date:……………………………

Please write any further comments here:

|  |
| --- |

**FOR OFFICE USE ONLY**

| **Procedure** | **Outcome of MDT Discussion**  **Date:** | **Outcome of further patient consultation if necessary.**  **Date:** |
| --- | --- | --- |
| Colposuspension |  |  |
| Synthetic Vaginal Mesh Tape |  |  |
| Natural Tissue Sling |  |  |
| Urethral bulking agent injection |  |  |

Clinician’s signature: ……………………………………………………Clinician’s name: …………………………………..…GMC No: ………: Date:……………………………

### Sources and acknowledgements

This information has been developed by the RCOG Patient Information Committee in collaboration with the British Society for Urogynaecology (BSUG). Below is a list of sources of evidence used.

1. [https://bsug.org.uk/budcms/includes/kcfinder/upload/files/BSUG%20National%20Report%20-%20Stress%20%20Incontinence%20Surgery%20in%20the%20UK%20(2008-2017).pdf](https://bsug.org.uk/budcms/includes/kcfinder/upload/files/BSUG National Report - Stress  Incontinence Surgery in the UK (2008-2017).pdf)
2. Ford AA, Rogerson L, Cody JD, Aluko P, Ogah JA. Mid-urethral sling operations for stress urinary incontinence in women. Cochrane Database of Systematic Reviews 2017, Issue 7. Art. No.: CD006375. DOI: 10.1002/14651858.CD006375.pub4
3. Lapitan M, Cody JD, Mashayekhi A. Open retropubic colposuspension for urinary incontinence in women. Cochrane Database of Systematic Reviews 2017, Issue 7. Art. No.: CD002912. DOI: 10.1002/14651858.CD002912.pub7
4. Rehman H, Bezerra CA, Bruschini H, Cody JD, Aluko P. Traditional suburethral sling operations for urinary incontinence in women. Cochrane Database of Systematic Reviews 2017, Issue 7. Art. No.: CD001754. DOI: 10.1002/14651858.CD001754.pub4
5. Dean N, Ellis G, Herbison G, Wilson D, Mashayekhi A. Laparoscopic colposuspension for urinary incontinence in women. Cochrane Database of Systematic Reviews 2017, Issue 7. Art. No.: CD002239. DOI: 10.1002/14651858.CD002239.pub3
6. Kirchin V, Page T, Keegan PE, Atiemo KOM, Cody JD, McClinton S, Aluko P. Urethral injection therapy for urinary incontinence in women. Cochrane Database of Systematic Reviews 2017, Issue 7. Art. No.: CD003881. DOI: 10.1002/14651858.CD003881.pub4
7. [Jha S](http://www.ncbi.nlm.nih.gov/pubmed/?term=Jha S%5BAuthor%5D&cauthor=true&cauthor_uid=21699671), [Ammenbal M](http://www.ncbi.nlm.nih.gov/pubmed/?term=Ammenbal M%5BAuthor%5D&cauthor=true&cauthor_uid=21699671), [Metwally M](http://www.ncbi.nlm.nih.gov/pubmed/?term=Metwally M%5BAuthor%5D&cauthor=true&cauthor_uid=21699671). Impact of incontinence surgery on sexual function: a systematic review and meta-analysis. [J Sex Med.](http://www.ncbi.nlm.nih.gov/pubmed/21699671) 2012 Jan;9(1):34-43[5].
